# Differential responses of invasive and native plants to warming with simulated changes in diurnal temperature ranges

**DOI:** 10.1093/aobpla/plx028

**Published:** 2017-07-24

**Authors:** Bao-Ming Chen, Yang Gao, Hui-Xuan Liao, Shao-Lin Peng

**Affiliations:** * State Key Laboratory of Biocontrol, Guangdong Provincial Key Laboratory of Plant Resources, School of Life Sciences, Sun Yat-Sen University, Guangzhou 510275, China

**Keywords:** Asymmetric warming, biomass allocation, global warming, plant invasion

## Abstract

Although many studies have documented the effects of global warming on invasive plants, little is known about whether the effects of warming on plant invasion differ depending on the imposed change in different diurnal temperature ranges (DTR). We tested the impact of warming with DTR change on seed germination and seedling growth of eight species in the family Asteraceae. Four of these are invasive (*Eupatorium catarium*, *Mikania micrantha*, *Biodens pilosa* var. *radiate*, *Ageratum conyzoides*) in China, and four are native (*Sonchus arvensis*, *Senecios candens*, *Pterocypsela indica*, *Eupatorium fortunei*). Four temperature treatments were set in growth chambers (three warming by 3 °C with different DTRs and control), and experiments were run to mimic wintertime and summertime conditions. The control treatment (*T_c_*) was set to the mean temperature for the corresponding time of year, and the three warming treatments were symmetric (i.e. equal night-and-day) (DTR_sym_), asymmetric warming with increased (DTR_inc_) and decreased (DTR_dec_) DTR. The warming treatments did not affect seed germination of invasive species under any of the conditions, but DTR_sym_ and DTR_inc_ increased seed germination of natives relative to the control, suggesting that warming may not increase success of these invasive plant species via effects on seed germination of invasive plants relative to native plants. The invasive plants had higher biomass and greater stem allocation than the native ones under all of the warming treatments. Wintertime warming increased the biomass of the invasive and wintertime DTR_sym_ and DTR_inc_ increased that of the native plants, whereas summertime asymmetric warming decreased the biomass of the invasives but not the natives. Therefore, warming may not facilitate invasion of these invasive species due to the suppressive effects of summertime warming (particularly the asymmetric warming) on growth. Compared with DTR_sym_, DTR_dec_ decreased the biomass of both the invasive and native plants, while the asymmetric summer warming treatments (DTR_inc_ and DTR_dec_) decreased the biomass of the invasive but not the native plants. In addition, wintertime DTR_inc_ did not enhance the biomass of all the plants relative to DTR_sym_. Our results were obtained in an unrealistic setting; the growth conditions in chambers (e.g. low light, low herbivory, no competition) are quite different from natural conditions (high light, normal herbivory and competition), which may influence the effects of warming on the seedling establishment and growth of both invasive and native plants. Nonetheless, our work highlights the importance of asymmetric warming, particularly in regards to the comparison with the effects of symmetric warming on both invasive and native plants. Conclusions regarding the effects of future warming should be made cautiously because warming with different DTRs may suggest different implications for invasion, and effects of warming may be different in different seasons.

## Teaser

We tested the impacts of unequal night-and-day warming on seed germination and seedling growth of invasive and native plants. Most warming treatments facilitated native seed germination but not that of invasives. The invaders performed better than the natives, and they allocated greater biomass to stems than the natives under all warming treatments. Compared with symmetric warming, both an increase and decrease in the asymmetric summer warming inhibited the growth of the invaders but not the natives, whereas the decrease in asymmetric winter warming inhibited the growth of both invasive and native plants. These findings highlight the importance of asymmetric warming in influencing plant invasion.

## Introduction

Mean temperatures have increased 1.0–1.6 °C globally, with daily minimum temperature (*T*_min_) showing a more rapid increase than daily maximum temperature (*T*_max_), resulting in a decrease of 0.066 °C per decade in the diurnal temperature range (DTR, *T*_max_ - *T*_min_) between 1950 and 2004 (Vose *et al.* 2005; IPCC 2013). Global warming is one of the salient features of global environmental change. In addition to the increases in mean annual temperatures, climate warming is asymmetric (i.e. warming with different DTRs) for different periods, seasons and continents (Liu *et al.* 2004; Wild *et al.* 2007; Rohde *et al.* 2013; Tan *et al.* 2015). For example, Rohde *et al.* (2013) and Wild *et al.* (2007) noted an increase in DTR globally since the mid-1980s. The DTR in India has increased as a result of a decrease in the minimum temperature (Rai *et al.* 2012). Zhou and Ren (2012) reported a significant decrease in DTR over mainland China of 0.15 °C per decade from 1961 to 2008. While an analysis conducted in southern China, the location of the present study, found an increase in DTR from 1984 to 2005 (Li and Chen 2009) and an increase in DTR from 1950 to 2100 was estimated with modelling study (Stone and Weaver 2003).

Seed germination percentages are often positively correlated with the temperature under which seeds are incubated (Graae *et al.* 2008), and diurnal fluctuations in temperature may stimulate seed germination (Thompson 1974; Thompson *et al.* 1977). Moderate summer warming substantially accelerated germination, and the magnitudes of the responses are species-specific (Milbau *et al.* 2009). In addition, plant growth is the balance of photosynthetic gains and respiratory losses. As photosynthesis occurs only during daytime and respiration occurs day and night, changes in DTR (a reduction or an increase in DTR) may alter the balance between photosynthesis and respiration. It has been reported that roughly half of daily net photosynthetic carbon fixation was re-released via plant respiration in the following evening (Ryan 1991), and the temperature response of leaf respiration was related to whole plant carbon, energy demands, and different leaf manipulations as well (Griffin *et al.* 2002). Turnbull *et al.* (2002) found that elevated night-time temperature increased photosynthetic capacity during the following light period through a respiratory-driven reduction in leaf carbohydrate concentration, indicating that increases in night-time minimum temperatures may have a significant influence on net plant carbon uptake. Decreased DTR (i.e. the occurrence of greater warming during the night than during the day) may increase night-time respiratory costs to a greater extent than it increases photosynthesis, resulting in reduced accumulation of organic matter by the plant (Phillips *et al.* 2011; Prasad and Djanaguiraman 2011). Therefore, it is meaningful to study the responses of plant seedling establishment to warming with both decrease and increase in DTR as a consequence of different night and day warmings.

Climate change and invasive alien species have threatened biodiversity throughout the twenty-first century (Vitousek *et al.* 1996; Bradley *et al.* 2010a; Vilà *et al.* 2011; Pyšek *et al.* 2012). Non-native invasive species and co-existing native species differ in many traits (Ordonez *et al.* 2010; Van Kleunen *et al.* 2010), and in general, invasive alien plants have greater dispersal, superior colonization ability, higher reproductive rate, rapid adaptive capacity and broad environmental tolerance (Dukes and Mooney 1999; Daehler 2003; Kriticos *et al.* 2003; Hellmann *et al.* 2008; Wolkovich and Cleland 2014). In many situations, climate warming may facilitate plant invasions and tend to increase invasion risk by accelerating physiological processes or growth of invasive species, or by increasing the competitive ability of invasive species (Alward *et al.* 1999; Hellmann *et al.* 2008; Walther *et al.* 2009; Bradley *et al.* 2010b; Kleinbauer *et al.* 2010; Verlinden and Nijs 2010; Wang *et al.* 2011).

Understanding whether invasive and native plants have systematically different responses to projected future temperature regimes could help predict future, risks from plant invaders. Although changes in DTR have been applied to models as an environmental factor that may predict shifts in the distributions of invasive plants (Cunze *et al.* 2013), few experimental studies have attempted to integrate changes in DTR and plant invasion. Recently, He *et al.* (2012) found that climate warming may facilitate invasion of the alien plant *Eupatorium adenophorum* by increasing its growth and stress-tolerance, and that night-warming increased the growth of the invasive species while it decreased that of a native congener. However, little is known about whether the impact of warming on invasive alien plants differs between symmetric warming and asymmetric warming.

Here, we hypothesized that, first, warming facilitates the biomass accumulation of invasive plants compared with native species. Second, warming with DTR change would benefit invasive plants more than symmetric warming since the balance between photosynthetic gains and respiratory losses can be altered more easily by asymmetric warming than by symmetric warming (with equal warming between daytime and night-time). In order to test these hypotheses, we specifically addressed the following questions: (i) do the responses to warming with different DTRs differ between plants native to a region and related introduced invaders, and (ii) does warming with DTR change have different effects on plant invasion compared with symmetric warming? We selected four invasive alien species (*Eupatorium catarium*, *Mikania micrantha*, *Biodens pilosa* var. *radiate*, *Ageratum conyzoides*) and four native species (*Sonchus arvensis*, *Senecios candens*, *Pterocypsela indica*, *Eupatorium fortunei*) from the Asteraceae family to examine their seed germination and seedling establishment, which are critical to population invasion and persistence and are important factors in species’ responses to global change (Williams *et al.* 2007). Because DTR (increase or decrease in DTR) differs in different regions and seasons, three warming patterns (with different DTR changes: increase in DTR, decrease in DTR, no change of DTR) and a control (normal temperature) of two seasons (winter and summer) were used to compare the responses of natives and non-native species to temperature changes.

## Methods

### Study species and seed collection

We chose eight of the most common species in Guangdong Province, southern China. Of these, four were invasive alien species (*M.micrantha*, *E.catarium*, *B.pilosa* var. *radiata*, *A.conyzoides*), and the rest were natives (*S.arvensis*, *Vernonia cinerea*, *Spilanthes paniculata*, *Eupatorium chinense*). Most of the seeds were collected from Xiaoguwei Island in Guangdong Province, southern China. The details of the eight species and seed source are given in Table 1.

**Table 1:** Characteristics of the eight species (four non-native invasive and four native species) used in the experiments. *Notes*: IS, invasive species; NS, native species. ∮Original place and temperature range within China where the species are distributed according to Scientific database of China plant species (http://www.plants.csdb.cn/eflora/default.aspx). All of the seeds were collected from Guangdong Province, southern China.

Plant origin	Species	Life form	Original place (∮)	Seed source (all are from Guangdong Province) and coordinates (latitude, longitude)	
IS	*Eupatorium catarium*	Perennial herb	South America	Near Guangdong Pharmaceutical University, Zhongshan city	22.52N, 113.38E
	*Mikania micrantha*	Perennial (climbing) herb	Tropical America	Qi’Ao Island, Zhuhai city	22.3N, 113.52E
	*Biodens pilosa var. radiate*	Annual herb	Tropical America	Xiaoguwei Island, Guangzhou city	23.16N, 113.23E
	*Ageratum conyzoides*	Annual herb	Middle South America	Xiaoguwei Island, Guangzhou city	23.16N, 113.23E
NS	*Sonchus arvensis*	Perennial herb	China	Xiaoguwei Island, Guangzhou city	23.16N, 113.23E
	*Senecios candens*	Perennial (climbing) herb	China	Nanling Mountain, Shaoguan city	24.84N, 113.62E
	*Pterocypsela indica*	Annual herb	China	Xiaoguwei Island, Guangzhou city	23.16N, 113.23E
	*Eupatorium fortunei*	Perennial herb	China	Xiaoguwei Island, Guangzhou city	23.16N, 113.23E

### Temperature settings

Guangdong Province has a typical subtropical climate characterized by hot, humid summers, with a mean annual temperature of 21.8 °C, annual relative humidity of 80 % and mean annual rainfall of 1786 mm. Four temperatures (control and three warming patterns) were set in four growth chambers (PQX-1000A, Ningbo Instruments, China) for wintertime and summertime experiments, respectively. The four temperatures included a control temperature and three warming patterns: symmetric warming (DTR_sym_) (i.e. equal night-and-day warming), increased diurnal temperature range (DTR_inc_) and decreased diurnal temperature range (DTR_dec_). The temperature settings were based on the standard monthly climate data of the past 30 years in Guangdong, China (the data were obtained from China Meteorological Data Sharing Service System). The average winter temperature is 14 °C, and the average summer temperature is 28 °C. The control temperature (*T_c_*, 19/11 °C for winter and 32/25 °C for summer) represents the seasonal (here, winter and summer were selected) averages of the daily maximum/minimum air temperatures (*T*_max_/*T*_min_). In addition, the annual average temperature in Guangdong will increase by 2.8 °C between 2071 and 2100 according to the assessment report on climate change of Guangdong (Composing Team for Assessment Report on Climate Change of Guangdong, 2007). Thus, in the study, the average temperature in the warming treatments (*T*_ave+_) (*T*_ave__+_ = (*T*_min+_ + *T*_max+_)/2) was increased by 3 °C relative to the average temperature of the control (*T*_ave_). Accordingly, symmetric DTR (DTR_sym_), a 3 °C decrease in DTR (DTR_dec_) and a 3 °C increase in DTR (DTR_inc_) were used as different changes in *T*_max_ and *T*_min_ (Stone and Weaver 2003; Lobell *et al.* 2007; Phillips *et al.* 2011). The details of the temperature settings for winter and summer are given in Table 2.

**Table 2:** Temperature settings. *T_c_* is control temperature. The temperatures in bold (presented in bold type) represent the diurnal temperature range (DTR). DTR_sym_, symmetric warming; DTR_inc_, increase in DTR; DTR_dec_, decrease in DTR.

Season	*T_c_* (*T*_max_/*T*_min_)	Warming treatment by + 3 °C (*T*_max+_/*T*_min+_)		
		DTR_sym_	DTR_inc_	DTR_dec_
Wintertime	19 °C/11 °C	19 + 3 °C/11 + 3 °C	19 + 4.5 °C/11 + 1.5 °C	19 + 1.5 °C/11 + 4.5 °C
		(22 °C/14 °C)	(23.5 °C/12.5 °C)	(20.5 °C/15.5 °C)
	**(8** °C**)**	**(8** **°C)**	**(11** **°C)**	**(5** **°C)**
Summertime	32 °C/25 °C	32 + 3 °C/25 + 3 °C	32 + 4.5 °C/25 + 1.5 °C	32 + 1.5 °C/25 + 4.5 °C
		(35 °C/28 °C)	(36.5 °C/26.5 °C)	(33.5 °C/29.5 °C)
	**(7** **°C)**	**(7** **°C)**	**(10** **°C)**	**(4** **°C)**

### Seed germination experiments

To examine the effects of DTR change on seed germination, we measured the seed germination of the four invasive and the four native species under simulated wintertime temperature changes. We did not test the effects of changes in DTR during summer on seed germination because few seeds germinate in the summer. The collected seeds were air-dried, cleaned and stored at room temperature before being used in the experiments. For each treatment, ten replicate plantings of 50 similar seeds of each species were seeded in pots (diameter: 12 cm; height: 11 cm) containing a 2:1:1 mixture of peat, river sand and vermiculite. The pots were watered every other day to maintain the soil–water content at 60 % (measured by the commonly used weight method) during seed germination. The seed pots were randomly placed in the four growth chambers that were programmed with the four temperature treatments (simulated temperature change of wintertime, Table 2). The accuracy of the chamber in manipulating temperature is ± 0.1 °C. Within the chambers, the light intensity was maintained at ∼220 μmol m^−2^ s^−1^ with a photoperiod of 8 h, and the relative humidity was maintained at ∼72 %. The conditions of all chambers were monitored and adjusted every day. Germination was defined as the emergence of the radical from the seed coat.

Germination was recorded daily; the recording ceased when no more seeds germinated.

(1) Germination Proportion(GP)(%) =germinated seeds/total seeds ×100

(2) $$Germination\;Index\;(GI)\;=\sum \left(\frac{Gt}{Dt}\right)$$

where *Gt* is the number of germinated seeds at incubation time *t* and *Dt* is the number of days in germination (1, 2, 3 … days). The germination index was the sum of the values of *Gt* by *Dt* from the first to the last day of the experiment.

### Plant growth experiments

Two experiments were conducted to simulate the warming conditions of wintertime and summertime. The experiments were performed in the growth chambers. Within the chambers, the light intensity was maintained at ∼220 μmol m^−2^ s^−1^ with a photoperiod of 8 h and 11 h, and the relative humidity was maintained at ∼72 and 79 % for simulated temperature changes of wintertime and summertime experiments, respectively. The conditions of all chambers were monitored and adjusted every day.

In the simulation experiments, a 2:1:1 mixture of peat, river sand and vermiculite was placed in basins and watered the day prior to sowing. For sowing, the seeds were evenly scattered on the surface of the mixed medium and covered with a thin layer of vermiculite. The basins were randomly placed in the growth chambers. Four growth chambers were used, one set as control (*T_c_*) and three to simulate the three warming treatments: symmetric warming (DTR_sym_), DTR_dec_ and DTR_inc_. The basins were watered daily to maintain the moisture content of the medium. When the seedlings of all the species under each temperature setting became sufficient in number for experimental replicates, uniform seedlings of each species under each temperature setting were transplanted into pots (diameter: 12 cm; height: 11 cm) containing the mixed medium. Each pot contained one individual plant. For each species in each treatment, 10 replicates (in the winter experiment) and 6 replicates (in the summer experiment) were randomly assigned to a single growth chamber. The pots were watered once every two days to maintain the water content of the medium at 60 % (determined by weight loss). Nutrient solution (i.e. 50 mL of a 0.1 % nutrient solution, Peters Professional (20 % N, 20 % P_2_O_5_, 20 % K_2_O, Scotts Company, USA) were added once every two weeks to provide sufficient nutrients to the plants. To maintain uniformity of the growth conditions, the pots were moved twice weekly within each chamber. In addition, to minimize chamber effects, the pots were switched from one chamber to another every two weeks, and each treatment had two rotations in the four chambers during the experiments (Leffler *et al.* 2013).

### Measurements

After four months’ growth under the temperature treatments, all the plant materials were harvested, and the total biomass per individual was measured after drying the harvested material for 72 h at 60 °C. Roots were separated from soil through soak, shake and rinse series with fine mesh sieves. The leaf mass ratio (LMR), stem mass ratio (SMR) and root mass ratio (RMR) were measured for each individual and averaged by species and treatment.

### Statistical analyses

All analyses were performed using IBM SPSS Statistics version 20.0 for Windows (IBM Corp., Armonk, NY, USA). General linear mixed models (GLMMs) were used to test the effects of temperature and plant origin on seed germination, plant biomass and root-shoot-ratio of the invasive and the native species. The main effect of temperature (*T_c_*, DTR_sym_, DTR_inc_, DTR_dec_) and plant origin (invasive or native) and their interactions were modelled as fixed factors, and species nested within origin was modelled as a random factor. In order to meet the assumptions of normality and variance homogeneity, outliers that deviate markedly from other observations had been removed from the dataset before the analyses were conducted. Decisions about outliers were made using ‘Data exploration’ command in SPSS, where extreme outliers were marked by a star in the boxplot. In order to further illustrate the interaction between species origin and temperature, the eight groups were generated by crossing the two species origins and the four temperature treatments. Turkey HSD post hoc tests were performed for these eight different groups, in which models, group was designated as a fixed factor, and species nested within group was designated as a random factor using GLMM command.

## Results

### Responses of seed germination to warming

**Figure 1.**
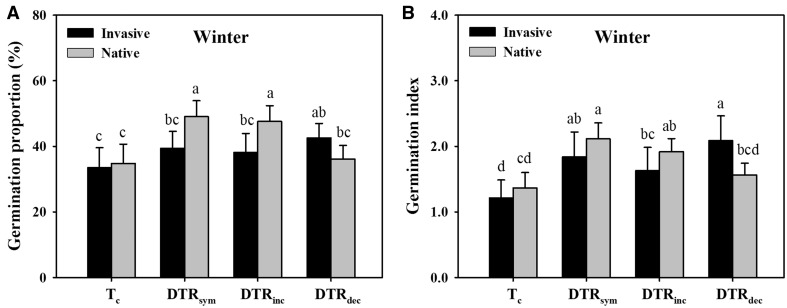
Germination proportion (A) and germination index (B) of invasive and native species under simulating variations of DTR of wintertime. Significant differences between each pair of treatments are presented using different symbols (*α* = 0.05). Error bars represent the mean + 1 SE.

Compared with the corresponding control (*T_c_*), the seed germination proportion of the native plants increased by 41.0 and 36.7 %, respectively under DTR_sym_ and DTR_inc_, while that of invasive plants increased by 27.3 % (Fig. 1A). The seed germination index showed similar responses to seed germination proportion (Fig. 1B). GLMMs analysis revealed significant temperature and temperature × origin interaction on seed germination, whereas plant origin (invasive vs. native) had no significant effect on seed germination (Table 3).

**Table 3:** Effects of temperature and origin (invasive vs. native) and their interactions on seed germination under simulated wintertime DTR changes. Seed germination was analysed using general linear mixed models with temperature (*T_c_*, DTR_sym_, DTR_inc_, DTR_dec_), origin (invasive plants, native plants) and their interactions as fixed factors and with species nested within the origin as a random factor. *Notes*: Numerator and denominator degrees of freedom are given for each effect (*F*-ratio subscript). Statistically significant values (*P* < 0.05) are presented in bold type.

Trait	Source	*F*	*P*
Germination proportion	Temperature	7.567(3,259)	**<0.001**
	Origin	0.053(1,5)	0.827
	Temperature × Origin	5.489(3,259)	**0.001**
Germination index	Temperature	22.644(3,259)	**<0.001**
	Origin	0.096(1,5)	0.770
	Temperature × Origin	7.885(3,259)	**<0.001**

### Response of plant biomass to warming

**Figure 2.**
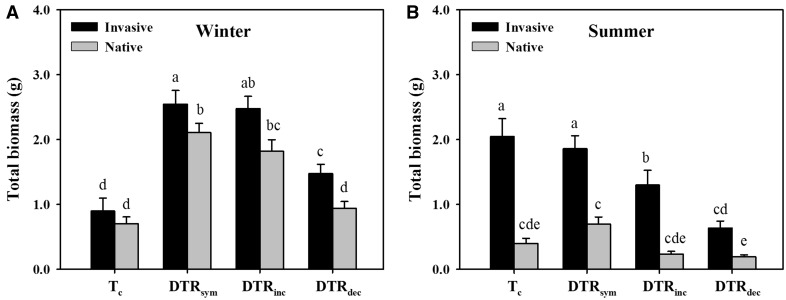
Total biomass of invasive and native species under simulating variations of DTR of wintertime (A) and summertime (B). Significant differences between each pair of treatments are presented using different symbols (*α* = 0.05). Error bars represent the mean + 1 SE.

Overall, the invasive plants accumulated greater biomass than the natives across all the summertime warming treatments and under wintertime DTR_sym_ and DTR_dec_. Both the invasive and the native plants grew better under DTR_sym_ than under DTR_dec_ in winter and summer (Fig. 2).

Temperature showed significant effects on both the invasive and native plants in winter and summer, and there were different responses to temperature change between invasive and natives in summer (Table 4). Under the simulated wintertime temperature change, as is shown in Fig. 2A, all three of the warming treatments (DTR_sym_, DTR_inc_ and DTR_dec_) increased the biomass of both the invasive and the native plants in comparison to the corresponding control (*T_c_*) except native plants under DTR_dec_. The results showed that DTR_dec_ of wintertime decreased the growth of both invasive and native plants relative to their corresponding DTR_sym_. The GLMMs analysis of the data for plants exposed to simulated wintertime temperature changes revealed a significant temperature and temperature × origin interaction on plant biomass (Table 4). Under simulated summertime temperature changes, the results showed that the biomass of the invasive plants decreased under DTR_dec_ and DTR_inc_ compared with the corresponding control (*T_c_*) and DTR_sym_, but not the native plants (Fig. 2B). The GLMMs analysis of the data for plants exposed to summertime temperature changes revealed a significant temperature, plant origin and temperature × origin interaction on plant biomass (Table 4).

**Table 4:** Effects of temperature, origin (invasive vs. native) and their interactions on plant growth and biomass allocation. Plant performances were analysed using general linear mixed models with temperature (*T_c_*, DTR_sym_, DTR_inc_, DTR_dec_), origin (invasive plants, native plants) and their interactions as fixed factors and with species nested within origin as a random factor. *Notes*: Numerator and denominator degrees of freedom are given for each effect (*F*-ratio subscript). Statistically significant values (*P*< 0.05) are indicated in bold type.

Trait	Source	Wintertime		Summertime	
		*F*	*P*	*F*	*P*
Biomass	Temperature	149.91(3,259)	**<0.001**	63.17(3,174)	**<0.001**
	Origin	0.39(1,6)	0.558	7.99(1,6)	**0.030**
	Temperature × Origin	3.99(3,259)	**0.008**	11.95(3,174)	**<0.001**
Leaf mass ratio	Temperature	101.29(3,260)	**<0.001**	4.59(3,175)	**0.004**
	Origin	0.02(1,6)	0.884	8.68(1,6)	**0.026**
	Temperature × Origin	0.56(3,260)	0.645	3.66(3,175)	**0.014**
Stem mass ratio	Temperature	20.89(3,222)	**<0.001**	19.60(3,131)	**<0.001**
	Origin	3.35(1,6)	0.141	5.10(1,6)	0.087
	Temperature × Origin	8.21(3,222)	**<0.001**	4.46(3,131)	**0.005**
Root mass ratio	Temperature	27.23(3,261)	**<0.001**	8.08(3,175)	**<0.001**
	Origin	1.24(1,6)	0.316	5.51(1,6)	0.057
	Temperature × Origin	8.87(3,261)	**<0.001**	2.08(3,175)	0.105

### Response of plant biomass allocation to warming

**Figure 3.**
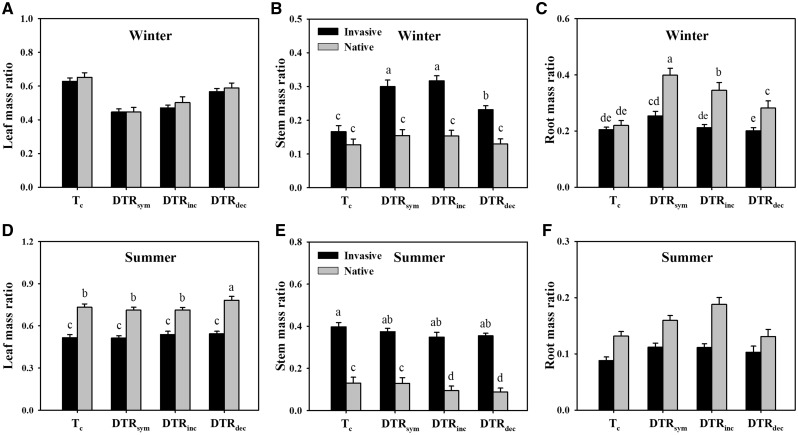
Biomass allocation of invasive alien plants and of native plants under temperature treatments of wintertime (A, B, C) and summertime (D, E, F). Significant differences between each pair of treatments are indicated by different symbols (*α* = 0.05). Error bars represent the mean + 1 SE.

Temperature had significant effects on the biomass allocation of both the invasive and the native plants, and there were significant differences between the invasive and the native plants in biomass allocation under summertime temperature changes (Table 4). The LMR of the native plants were higher than those of invasive plants under all the treatments of summertime, while there were no significant differences in LMR between invasive and native plants under wintertime (Fig. 3A, D). The SMR of the invasive plants was much higher than the native ones, and wintertime warming increased the SMR of the invasive plants (Fig. 3B, E). There were no significant effects of plant origin on RMR under both wintertime and summertime (Fig. 3C, F, Table 4). DTR_sym_ and DTR_inc_ of wintertime increased the RMR of the native plants in contrast to the corresponding *T_c_* (Fig. 3C).

## Discussion

Invasive plant populations have been shown to germinate more quickly and to exhibit higher growth rates than native species (Erfmeier and Bruelheide 2005). Considering merely the impacts of warming patterns on the invasive plants *per se*, our results showed that the warming treatments did not affect germination of invasive species under any of the conditions, while wintertime warming increased and asymmetric summer warming decreased the biomass accumulation of the invasive species. In addition, wintertime warming enhanced stem allocation of the invasive plants, while summertime warming did not alter the stem allocation. These results suggest that warming may not facilitate the success of these invasive plant species due to the opposing impacts of warming in winter and summer. Although in many modelling studies of invasive alien plants and expansion under simulated temperature changes, native plants are not used as controls (Kleinbauer *et al.* 2010; Plank *et al.* 2016), climate warming sometimes has different effects on alien plant species compared with their closely related native counterparts (Verlinden and Nijs 2010). Therefore, we compared the impacts of symmetric and asymmetric warming between the invasives and the natives to evaluate the effects of warming on plant invasion. Our results showed that DTR_sym_ and DTR_inc_ facilitated seed germination of the natives relative to control, but had no significant effect on the invasives. This suggests that warming may not increase success of these invasive plant species via effects on seed germination of invasive plants relative to native plants. Invasive plants produced more biomass than native plants under most of the treatment conditions (Fig. 2). The wintertime warming (DTR_sym_, DTR_dec_ and DTR_inc_) almost always increased the biomass of both invasive and native plants. DTR_dec_ increased the biomass of the invasives more than that of the natives in winter temperature regimes, but suppressed growth of invasives more than natives in summer as did DTR_inc_. Overall, this provides at best very weak support for our hypothesis that warming would facilitate the biomass accumulation of invasive plants more than that of native species. Amongst the three warming patterns, the biomass of both invasive and native plants was the lowest under DTR_dec_. The higher night-time temperature of DTR_dec_ than those of DTR_sym_ and DTR_inc_ may increase night-time respiratory costs, resulting in reduced accumulation of organic matter (Manunta and Kirkham 1996; Phillips *et al.* 2011). In the present study, the biomasses of the invasive and the native plants under DTR_dec_ were lower than those observed under DTR_sym_ and DTR_inc_ in wintertime while only the invasives appeared to be lower in summertime, which is consistent with the results of previous studies of crops and natural ecosystems (Nemani *et al.* 2001; Peng *et al.* 2004, 2013). This finding is also supported by the results that night warming decreased the biomass of both the invasive alien plant *E.adenophorum* and a native congener *E.chinense* (He *et al.* 2012). Furthermore, wintertime DTR_dec_ decreased the stem allocation of invasive plants relative to DTR_sym_. Stem growth is thought to enhance plant fitness by increasing light interception and to enhance climbing capacity of lianas/vines. These results suggest that DTR_dec_ may reduce the risk of the invasive species relative to DTR_sym_.

In addition to the global trend of a reduction of DTR, DTR trends differ amongst regions. An increase in DTR was found from 1984 to 2005 (Li and Chen 2009) and from 2003 to 2012 (Alkama and Cescatti 2016), and an increase in DTR from 1950 to 2100 was estimated with modelling study (Stone and Weaver 2003) in southern China (the location of the present study). Thus, the results of DTR_inc_ obtained in the present study could be more accurate in estimating the performance of the invasive plants in the future. Comparing the results of warming DTR_inc_ with those of DTR_sym_, we found warming DTR_inc_ may not increase the risk of the invasive species through seed germination as there was no significant difference between DTR_sym_ and DTR_inc_. The summertime DTR_inc_ decreased the biomass accumulation of invasive plants relative to DTR_sym_, while wintertime DTR_inc_ had no significant effects on both invasive and native plants. Compared with symmetric warming, wintertime DTR_dec_ and DTR_inc_ led to less decrease in biomass of invasive plants than in native ones. This did not support our hypothesis that warming with DTR change may facilitate invasive plants more greatly than symmetric warming.

Some invasive plants may have a greater capacity to shift their physiological optimum to a range that is favourable in the new climate (Richards *et al.* 2006; Maron *et al.* 2007). The predicted global warming could provide new opportunities for introductions to areas where introduced species were not able to survive (Walther *et al.* 2009). In the present study, most of the invasive plants originate from tropical America, where the mean temperature of winter is higher than that of the introduced region (southern China). Hence, wintertime warming may facilitate the invasive alien species from habitats in warmer regions (their originated tropical America) to new areas with colder winter conditions (the region of the present study subtropical area), which is supported by our results that wintertime warming increased the biomass of the invasive plants (Fig. 2A) and wintertime warming facilitated more biomass allocation to the stems of the invasive plants (Fig. 3B). However, summertime warming decreased the biomass of the invasive plants, which may inhibit the invasion of the alien plants (Fig. 2B). It should be noted that the growth conditions in chambers are different from natural conditions. In the chambers, the light is lower than in nature, most of the herbivores and soil microbes are excluded compared with natural conditions, and competition with other plants is reduced or eliminated.

The work to evaluate the impact of warming on plant invasion is complicated because warming with different DTR changes in different regions and during different periods (seasons) can produce different results, making it harder to determine invasive plant expansion at large scale rather than at more local regions in the future. Furthermore, the present study compared the relative change between invasive and native plants, but the effects resulted from monoculture may be different from the real competition between the invasive and native plants. The success of invasions is closely linked to the co-evolution and competitive outcomes between invasive plants and native plants (Callaway and Maron 2006; Verlinden *et al.* 2014). Therefore, more attention should be paid to the interactions between the invasive plants and the co-existing native plants under warming with different DTRs in field study or in the conditions closer to natural conditions in the future.

## Conclusions

Our results indicate that the warming may not alter success of these invasive plant species via effects on seed germination. Most wintertime warming treatments increased the biomass of both the invasive and native plants, whereas most summertime warming treatments decreased the biomass of the invasives. This indicates that the different effects of warming in different seasons should be taken into account when we estimate the effects of warming on plant invasion. Furthermore, warming with DTR change had different impacts relative to DTR_sym_, and there were some different impacts on plant growth between DTR_dec_ and DTR_inc_. Therefore, conclusions regarding the effects of future warming should be made cautiously as warming with different DTRs in different seasons may suggest different implications for invasion. Our work highlights the importance of asymmetric warming, particularly in regards to the comparison with the effects of symmetric warming on both invasive and native plants in winter and summer.

## Sources of Funding

The study was supported by the National Natural Science Foundation of China (31670479), the Natural Science Foundation of Guangdong (2016A030313287), Science and Technology Planning Project of Guangzhou (201607020027) and Training Program Foundation for the Excellent Youth Scholars by Educational Commission of Guangdong Province (Yq2013132).

## Contributions by the Authors

B.M.C, Y.G. and S.L.P. conceived and designed the experiments. Y.G. performed the experiments. B.M.C, Y.G. and H.X.L. analysed the data. S.L.P. contributed reagents and analysis tools. B.M.C. and Y.G. wrote the paper.

## Conflict of Interest Statement

None declared.
